# Precollapse femoral head avascular necrosis after combined surgical-medical management

**DOI:** 10.6026/973206300214654

**Published:** 2025-12-15

**Authors:** Mahendra Solanki, Abhishek Pal, Swapnil Mathur

**Affiliations:** 1 Department of Orthopaedics, MGM Medical College, Indore, Madhya Pradesh, India

**Keywords:** Femoral head, Avascular necrosis, Core Decompression, Surgical, Bone Transplantation, Precollapse stage, Harris Hip Score

## Abstract

Avascular necrosis of the femoral head occurs due to loss of blood supply leading to osteocyte death and progression to collapse if not treated early. Hence, we followed 60 patients (80 hips) with precollapse AVN (Modified Ficat-Arlet stage I and IIA) who underwent core decompression with autologous cancellous iliac crest grafting plus a fixed pharmacotherapy protocol for 48 weeks. Harris Hip Score improved serially, with unilateral hips reaching 87.5% and bilateral hips 90% good to excellent outcomes at final follow up and no hip remaining in poor category. Most hips were Stage I and idiopathic was the common etiology and the results support that combined surgical plus medical management gives meaningful short-term functional benefit when started early.

## Background:

Avascular necrosis (AVN) of femoral head is a disabling disorder caused by loss of blood supply that leads to osteocyte death and gradual collapse of the head [1, 2]. Progressive collapse results in hip dysfunction and if untreated secondary osteoarthritis develops [1]. The antero-superior quadrant of femoral head carries maximum load and is the commonest site of AVN [3]. Early detection is key for good outcome. MRI is most sensitive in early disease while X-ray is useful once collapse and degenerative changes appear [4]. AVN shows wide international variation, accounting for only 5-18% of primary THAs in the US but more than 50% in Indian registries, making it the leading cause of hip replacement in India compared to osteoarthritis in the West [5, 6]. The disease shows clear age and sex variation, with highest incidence in men aged 25-44 and in women aged 55-75, and overall a male predominance with nearly 4:1 ratio [7]. Corticosteroid use explains almost 40% non-traumatic cases and alcohol intake nearly 30% also Frequency has increased with more steroid use and better imaging [8]. Non-traumatic etiology causes include corticosteroids, alcohol, sickle cell disease, post-COVID vascular injury and idiopathic [6, 7, 8-9]. COVID-19 associated hypercoagulable state and steroid therapy further increase risk [10]. Management depends on stage. Pharmacological agents like bisphosphonates, anticoagulants and supportive supplements may help in early disease. Surgical options include core decompression, bone grafting, vascularized grafts and finally total hip arthroplasty for advanced collapse [11, 12]. Harris Hip Score (HHS) is widely used to assess functional outcome of these interventions [13]. Therefore, it is of interest to preserve femoral head and delay arthroplasty especially in younger patients.

## Materials and Methods:

This prospective observational study included 60 patients with 80 hips of early stage AVN. Total follow up was 48 weeks. Patients more than 18 years of age with Modified Ficat-Arlet stage I and IIA were taken.

## Inclusion criteria:

All OPD patients with AVN femoral head, stage I and IIA, age above 18 years.

## Exclusion criteria:

Patients with stage ≥IIB, traumatic AVN, associated spine or knee pathology, sickle cell anemia, infection, autoimmune, neurovascular and neurodegenerative disease were excluded.

## Sampling:

Sequential method was used. First 46 eligible patients reporting to OPD were taken and then extended to reach final sample size of 60.

## Clinical and imaging:

All patients underwent detailed history, clinical exam, routine pre-anesthetic blood tests and sickle test. X-ray pelvis with both hips AP and frog-leg lateral was done. Early AVN may appear normal on X-ray but later stages show sclerosis, cystic change or crescent sign [14]. MRI of hip was performed in all patients and is most sensitive for early ischemic change [15].

## Staging:

AVN was staged according to Modified Ficat-Arlet classification. This version combines X-ray, MRI and biopsy features to improve accracy for prognosis and treatment planning [15].

## Study protocol:

[1] Surgical procedure: Core decompression with autologous cancellous bone grafting under standard technique.

[2] Pharmacotherapy: Aspirin 75 mg daily for 2 weeks, calcium and vitamin D3 supplements for 48 weeks, vitamin C daily for 48 weeks, alendronate 70 mg weekly starting after 2 weeks.

[3] Post-op: Partial to full weight bearing as per protocol, suture removal at 2 weeks.

[4] Follow up: Clinical assessment and Harris Hip Score at 6, 12, 24 and 48 weeks.

## Surgical technique:

Core decompression was done using standard approach as in Figure 1 (see PDF). Multiple drill holes were made to reduce intraosseous pressure and autologous cancellous bone graft from iliac crest was packed in the tract to give structural support and enhance healing. Post-operative protocol included aspirin 75 mg once daily for 2 weeks, calcium daily for 48 weeks, vitamin D3 60,000 IU weekly for 48 weeks, vitamin C-500 mg daily for 48 weeks and alendronate 70 mg weekly started after 2 weeks. High protein diet was advised, pain killers and proton pump inhibitors given when needed. Follow up protocol was different for unilateral and bilateral cases. In unilateral cases partial weight bearing was allowed from day 1 till 2 weeks then full weight bearing as tolerated. In bilateral cases non-weight bearing for first 2 weeks, partial till 6 weeks, then full as tolerated. First follow up was done at 2 weeks for suture removal. Functional outcome was assessed using Harris Hip Score at 24 and 48 weeks post operatively.

## Results:

A total of 60 patients with 80 hips were studied. Mean age was 30 years (range 19-45) with male predominance 58.3% (Table 1 - see PDF). Unilateral AVN was more common seen in 66.7% while bilateral in 33.3%. Idiopathic cause was most frequent (45%) followed by corticosteroid use, alcohol and post-COVID infection (Table 2 - see PDF). According to Modified Ficat-Arlet classification majority hips were in stage I (56.2%) and rest in stage IIA (43.8%) showing patients mainly presented in precollapse stage (Table 3 - see PDF). Harris Hip Score improved consistently at each follow up. In unilateral cases mean HHS increased from 70.9 preoperatively to 87.4 at 48 weeks. Poor category reduced from 19 to none and excellent outcome reached 20 hips at final follow up (Table 4 - see PDF, Figure 2 - see PDF). In bilateral cases mean HHS increased from 70.4 to 86.6 at 48 weeks. Poor cases reduced from 10 to none and excellent outcome was noted in 8 hips at final follow up (Table 5 - see PDF, Figure 2 - see PDF). Overall at 48 weeks 88.3% hips achieved good to excellent functional outcome and no patient remained in poor category (Table 6 - see PDF).

## Discussion:

Our study showed that core decompression with autologous cancellous bone grafting plus pharmacotherapy in early AVN gave consistent improvement in Harris Hip Score over 48 weeks. At final follow up 88.3% of hips achieved good to excellent outcomes and no patient remained in poor category. This is close to findings of Steinberg *et al.* where early stage hips had far better survivorship with only 14% of small lesions requiring THA while larger lesions failed in 42-48% [16]. Nishii *et al.* also observed collapse rate fell to 5% with alendronate compared to 46% without drug, highlighting role of adjuvant pharmacotherapy [17]. Agarwala from India reported similar success with bisphosphonate combination in young post-chemotherapy patients [18]. Other augmentation procedures are also being tested. Villa *et al.* in a systematic review noted that decompression with bone marrow cells reduced collapse risk though THA rates were unchanged [19]. Gadkari showed decompression with intraosseous ibandronate gave faster recovery than decompression alone [20]. Ansari reported favorable mid-term preservation with single incision autograft technique, while Akhta *et al.* showed arthroscopic assisted decompression has promising short-term function [21, 22]. Still outcomes are not uniform as Andronic *et al.* found in 1134 hips nearly 38% failed and needed THA within 26 months after simple decompression [23]. Wei *et al.* in a large multicentre series showed 52.4% overall failure and highlighted steroid use, large necrotic angle and longer disease duration as major predictors [24]. Demirel *et al.* studying lupus patients showed poor long term survival with only 31% hips preserved at 10 years and 12.5% at 15 years [25]. These negative reports show that core decompression is not a universal solution and long term preservation cannot be assured. In mixed stage cohorts THR gave 90% excellent and 10% good HHS showing replacement still gives the most reliable pain relief once the head has collapsed [26]. Our cohort was mainly young idiopathic and steroid-related patients who presented in precollapse stage I and IIA explaining better outcomes. Both unilateral and bilateral cases improved though unilateral hips recovered faster a trend also seen by Talmaç and Mulyadi where smaller lesions had higher chance of hip survival [27, 28]. Role of supplements and bisphosphonates may also have helped. Barrios-Garay proved vitamin C supports bone healing, and Agarwala emphasized bisphosphonates delay collapse [18, 29]. Taken together the findings indicates that decompression plus grafting combined with medical therapy is most effective when started early but patient selection and long term monitoring are essential. The strength of this study is that it was prospective with same surgical technique and fixed drug protocol in all cases. We had sixty patients and eighty hips which is bigger than many single centre reports. Harris Hip Score was checked at different follow ups for one year and only stage I and IIA hips were taken so advanced cases did not confuse the results. The main limitation is that it was single centre and follow up only till forty eight weeks so late collapse and conversion to arthroplasty could not be seen. Imaging changes were not measured in detail and there was no control group for direct comparison with conservative care. In future longer follow up beyond five years is needed to see true hip survival. Multicentric trials should compare simple decompression with augmentation like bone marrow cells, vascularized grafts and bisphosphonates. Patient selection based on necrotic angle and etiology and cost effectiveness in Indian set up should also be studied.

## Conclusion:

We show that early stage AVN of femoral head treated with core decompression, cancellous grafting and pharmacotherapy leads to consistent functional improvement within one year. Unilateral hips recovered faster than bilateral but both groups reached more than 85% good to excellent outcome at final follow up. Idiopathic and steroid related AVN dominated in young patients which explains better short term response compared to advanced disease. Early diagnosis and timely combined treatment remain key to preserve the native hip and delay arthroplasty in this population.
